# Supplementary material for: Disinfecting Action of Gaseous Ozone on OXA-48-Producing Klebsiella pneumoniae Biofilm In Vitro
Source: Int J Environ Res Public Health. 2022 May 19;19(10):6177. doi: 10.3390/ijerph19106177 (PMC9140702; doi:10.3390/ijerph19106177)
Supplement: Supplementary file 1 [file ijerph-19-06177-s001.zip › ijerph-1695574-supplementary.pdf]

Disinfecting Action of Gaseous Ozone on OXA-48-Producing *Klebsiella pneumoniae* Biofilm In Vitro

Kaća Piletić [1], Bruno Kovač [1], Marko Perčić [2], Jure Žigon [3], Dalibor Broznić [4], Ljerka Karleuša [5], Sanja Lučić Blagojević [6], Martina Oder [7] and Ivana Gobin [1,*]

[1] Department of Microbiology and Parasitology, Faculty of Medicine, University of Rijeka, 51000 Rijeka, Croatia; kaca.piletic@student.uniri.hr (K.P.); bruno.kovac@student.uniri.hr (B.K.)
[2] Faculty of Engineering & Centre for Micro- and Nanosciences and Technologies, University of Rijeka, 51000 Rijeka, Croatia.; marko.percic@uniri.hr
[3] Department of Wood Science and Technology, Biotechnical Faculty, University of Ljubljana, 1000 Ljubljana, Slovenia; jure.zigon@bf.uni-lj.si
[4] Department of Medical Chemistry, Biochemistry and Clinical Chemistry, Faculty of Medicine, University of Rijeka, 51000 Rijeka, Croatia; dalibor.broznic@uniri.hr
[5] Department of Physiology and Immunology, Faculty of Medicine, University of Rijeka, 51000 Rijeka, Croatia; ljerka.karleusa@uniri.hr
[6] Faculty of Chemical Engineering and Technology, University of Zagreb, 10000 Zagreb, Croatia; slucic@fkit.hr
[7] Department of Sanitary Engineering, Faculty of Health Sciences, University of Ljubljana, 1000 Ljubljana, Slovenia; martina.oder@zf.uni-lj.si
* Correspondence: ivana.gobin@uniri.hr

Table S1. Antibiotic resistance/sensitivity pattern of the *K. pneumoniae* isolates.

Isolate	Kp strain14	Kp strain 15	Kp strain 16	Kp strain 33	Kp strain 34	KpATCC 700603	KpNCTC 13442
**Specimen**	**Urine**	**Urine**	**Urine**	**Blood**	**Urine**	**Urine**	**Unknown**
**Antibiotics**	**Susceptibilities of *K. pneumoniae* isolates**						
**AMX**	R	R	R	R	R	R	R
**AMX-C**	R	R	R	R	R	R	R
**TZP**	R	R	R	R	R	S	R
**CEX**	R	R	R	R	R	R	R
**CXM**	R	R	R	R	R	R	R
**CRO**	R	R	R	R	R	R	S
**CAZ**	R	R	R	R	R	R	R
**CDP**	R	R	R	R	R	R	R
**CFIX**	R	R	R	R	R	R	S
**CFMP**	R	R	R	R	R	R	S
**IMP**	R	S	I	R	I	S	S
**MEM**	R	I	R	R	R	I	R
**ETP**	R	R	R	R	R	S	R
**GEN**	R	S	R	R	R	R	S
**AMK**	S	S	S	R	S	S	S
**NRFX**	R	R	R	R	R	S	R
**CIX**	R	R	R	R	R	I	R
**SXT**	R	R	R	R	R	R	R
**TC**	R	R	R	S	R	R	R
**COL**	S	S	S	R	S	S	S
**COL MIC microdillution** μg/mL	0.500	0.250	0.500	64.00	0.250	0.500	0.125

AMX – amoxicilin, AMX-C – amoxicilin clavulanic acid, AMP – ampilicin, TZP – piperacilin tazobactam, CEX – cephalexin, CXM – cefuroxime, CRO – ceftriaxone, CAZ – cephtazidime, CDP – cefpodoxime, CFIX – cefixime, CFMP – cefepime, IMP – imipenem, MEM – meropenem, ETP – ertapenem, GEN – gentamicin, AMK – amikacin, CIX – ciprofloxacin, NRFX – norfloxacin, SXT – sulfamethoxazole trimethoprim, TC – tigecycline, COL – colistin, S – sensitive, I – sensitive with prolonged exposure, R – resistant, MIC – minimal inhibitory concentration

Table S2. Biofilm inhibition of all *K. pneumoniae* strains. Results are shown in percentages (%).

Bacteria	%Kp strain 14	%Kp strain 15	%Kp strain 16	%Kp strain 33	%Kp strain 34	%Kp ATCC 700603	%Kp NCTC 13442
**CFU/cm²**	97.8	98.88	98.73	98.66	99.09	99.36	99.76
**RLU**	78.36	79.79	84.76	75.85	76.23	85.93	88.15
**A/600nm**	40.98	38.72	35.87	48.61	44.11	22.14	34.07
